# Upper Extremity Fractures in Children—Comparison between Worldwide, Romanian and Western Romanian Region Incidence

**DOI:** 10.3390/children7080084

**Published:** 2020-07-29

**Authors:** Ovidiu Adam, Florin George Horhat, Elena Amaricai, Vlad-Laurentiu David, Zoltán Derzsi, Eugen Sorin Boia

**Affiliations:** 1 Department of Pediatric Surgery and Orthopedics, “Victor Babes” University of Medicine and Pharmacy Timisoara, Eftimie Murgu Street No. 2, 300041 Timisoara, Romania; adamovidiu29@yahoo.com (O.A.); boiaeugen@yahoo.com (E.S.B.); 2 Department of Microbiology, “Victor Babes” University of Medicine and Pharmacy Timisoara, Eftimie Murgu Street No. 2, 300041 Timisoara, Romania; 3 Department of Rehabilitation, Physical Medicine and Rheumatology, “Victor Babes” University of Medicine and Pharmacy Timisoara, Eftimie Murgu Street No. 2, 300041 Timisoara, Romania; amaricai.elena@umft.ro; 4 Department of Pediatric Surgery and Orthopedics, George Emil Palade University of Medicine, Pharmacy, Science and Technology of Targu Mures, Gh. Marinescu Street No. 38, 540139 Targu Mures, Romania; zoltanderzsi@yahoo.com

**Keywords:** upper extremity fractures, children, incidence

## Abstract

(1) Background: Fractures represent a significant part of all pediatric injuries, with distal forearm fracture being the most common fracture type in children. (2) Methods: In this comparative, epidemiological study we collected fracture incidence data from the scientific literature and compared it to real-world data extracted from the Romanian national and regional hospital database. In order to collect information on the epidemiology of upper extremity fractures in children, we conducted a systematic literature review on Medline, via PubMed. Extracted incidence data were stratified by fracture location, age or age interval and gender. Nationwide and Western Region incidence values were calculated for different fracture locations of the upper extremity using data extracted from a centralized hospital database. Incidence values were calculated using the mid-2018 census data. The search was restricted to the pediatric population. (3) Results: Incidence values for upper arm fractures nationwide and for Western Region were 54.83/100,000 person-years and 64.79/100,000 person-years, respectively. Forearm fractures had an incidence of 139.77/100,000 person-years and 139.56/100,000 person-years, respectively. The overall incidence of upper extremity fractures nationwide and for the Romanian Western Region were 206.02/100,000 person-years and 220.14/100,000 person-years, respectively. (4) Conclusions: Incidence of upper extremity fractures in the pediatric population varies according to the analyzed data. The calculated incidence depends on the site of fractures, assessed population (worldwide, Romanian population or regional-Western part of Romania) or patients’ age.

## 1. Introduction

**Figure 1:**
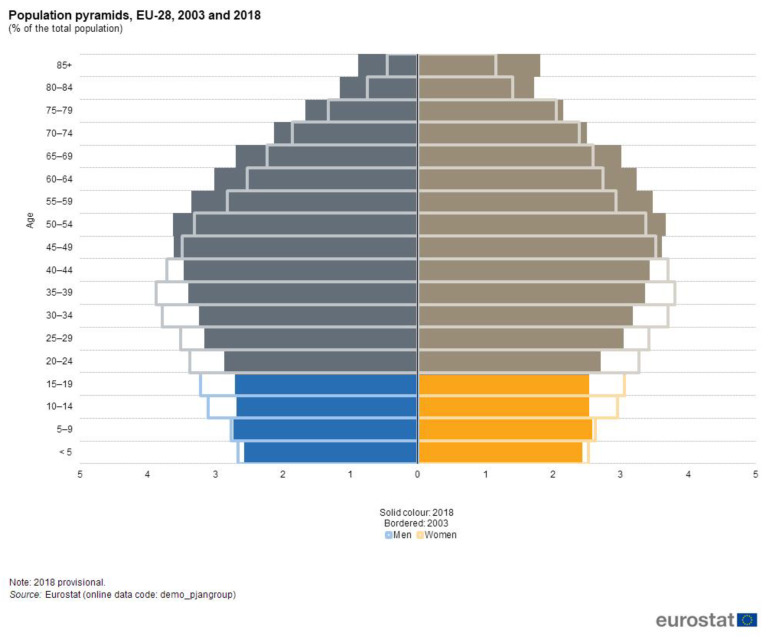
EU28 population structure comparison 2003–2018 [8].

Fractures are frequently met in children, representing 10% to 25% of all pediatric injuries [1,2,3,4]. The distal forearm fracture is the most common type of fracture in childhood and adolescence [5]. Around one-third of all children suffer at least one fracture before the age of 17 years [6]. The most common mechanism of fractures is represented by falls [7]. The European pediatric population, as well as the Romanian, has an overall decreasing trend according to Eurostat (Figure 1) [8]. In the meantime, winter sports, extreme sports, indoor and outdoor sports equipment, team sports and urban sports are becoming increasingly popular [9]. This means that even though there is an overall decreasing trend of the European pediatric population, the incidence of fracture in children is not decreasing and there still is a significant burden to the European countries’ health systems.

Demographic trends and disease incidences are used as an instrument in healthcare decision- making at national, regional and hospital level. Recently published upper extremity fracture incidence data for Romania is very limited and even more limited at the regional level (Romanian Western Region, RO42-NUTS classification [10]). The objectives of our study were to calculate the upper extremity fracture incidence values nationwide and in the Romanian Western Region, and to perform a systematic literature search capturing similar incidence values from the scientific research.

## 2. Materials and Methods 

In this comparative, epidemiological study, we collected fracture incidence data from the scientific literature and compared it with real-world data extracted from the Romanian national and regional hospital database.

### 2.1. Data Extraction from the Scientific Literature

A systematic literature review was performed in order to capture incidence information published in the last five years in the scientific literature regarding the upper extremity fractures in children. The systematic literature search was performed on 27 January 2020, using the Medline database (via PubMed). Our search was limited to English language papers published in the last five years (2015–2020). No geographical restrictions were applied in order to provide a comprehensive picture of the up-to-date fracture incidence. The search strategy used a combination of search strings, allowing the capture of relevant keywords and synonyms. Database search was performed using the following algorithm: “((upper extremity[Title/Abstract] OR forearm[Title/Abstract] OR humerus [Title/Abstract] OR ulna[Title/Abstract] OR radius[Title/Abstract]) AND fracture*[Title/Abstract]) AND (incidence[Title/Abstract] OR epidemio*[Title/Abstract]) AND (child*[Title/Abstract] OR pediatr*[Title/Abstract] OR paediatr*[Title/Abstract]) AND “last 5 years”[PDat]”.

The literature search resulted in 132 hits. Titles and abstracts were collected and a title/abstract screening was performed by two independent investigators selecting relevant papers. Disagreements between the two researchers were resolved by the principal investigator. The following exclusion criteria were applied: (1) duplicate; (2) not reporting original data (letter, comment, not systematic review); (3) case report and case series; (4) no upper extremity fracture data (or other types of injuries, e.g., joint and soft tissue injury present); (5) no epidemiologic (incidence) data. 

**Figure 2:**
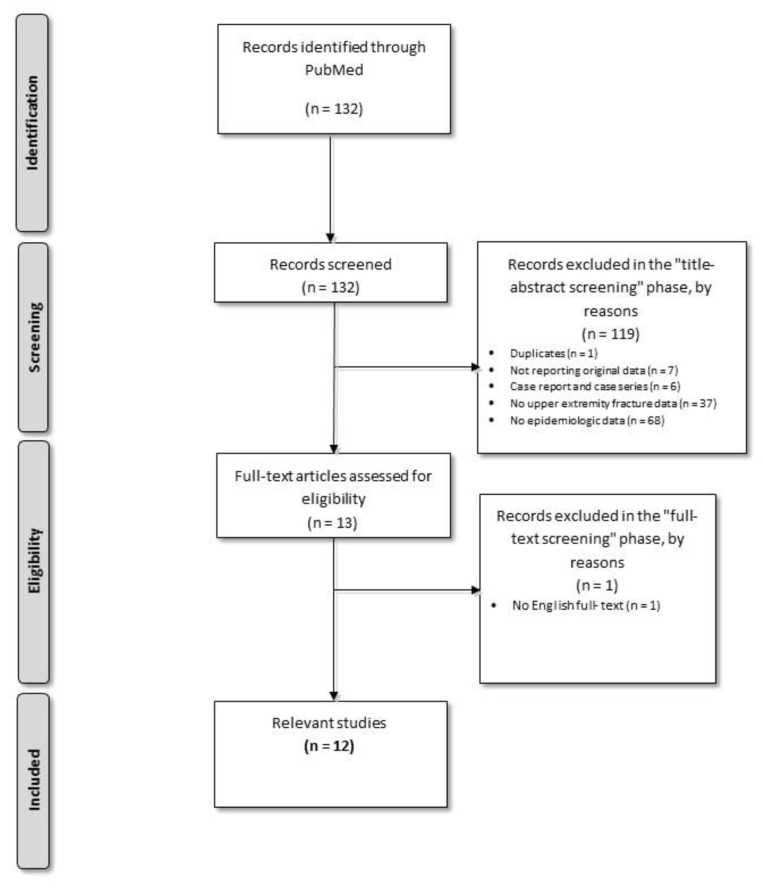
Systematic literature review flow diagram.

A total of 119 studies were excluded in this phase. Figure 2 shows the study analysis flow diagram. The title/abstract screening phase resulted in 13 relevant papers. One paper did not have an English language full-text and was therefore excluded.

The included articles were analyzed in full-text. The following data were extracted by an independent investigator: fracture location, fracture incidence, patient age (or age interval) and patient gender. Extracted data were double-checked and validated by a second investigator. The results were stratified by fracture location, age or age interval and gender. Stratification based on fracture location was as follows: upper arm, forearm, upper limb (location not specified). 

### 2.2. Calculation of Nationwide and Regional Level Incidence Data

Using the ICD-10-AM, 3rd edition diagnosis codes as search terms, a database search was performed using the centralized hospital database comprising anonymized inpatient data. Both national and regional incidence data for upper extremity fractures were extracted. The search was restricted to a one-year interval (2018) and to a population younger than 18 years. Both male and female patients were included. For upper arm fracture, the following ICD-10 codes were used: S42.20, S42.21, S42.22, S42.23, S42.24, S42.29, S42.3, S42.40, S42.41, S42.42, S42.43, S42.44, S42.45 and S42.49. For forearm fracture the following ICD-10 codes were applied: S52.00, S52.01, S52.02, S52.09, S52.20, S52.21, S52.31, S52.4, S52.6, S52.10, S52.11, S52.12, S52.19, S52.30, S52.50, S52.51, S52.52 and S52.59. For upper limb fractures (not otherwise specified), the following ICD-10 codes were used: M84.32, M84.33, M84.42, M84.43, S52.7, S52.8, S52.9 and S51.81.

An overall incidence including all previously mentioned ICD-10 codes was also calculated, representing the combined incidence for upper arm, forearm and upper limb (not otherwise specified) fractures. Census results from 2018 were used as national and regional population data; incidence values were calculated per 100,000 person-years.

## 3. Results

Outcomes of the systematic literature review included the incidence of upper extremity fractures in children, stratified by fracture location. Incidence data were extracted from relevant studies for the following fracture locations: upper arm (humerus, humerus shaft, proximal humerus and distal humerus), forearm (shaft, diaphyseal, distal), radius/ulna (proximal, diaphyseal, distal and total), radius (distal, shaft), ulna shaft and upper limb (location not specified).

### 3.1. Upper Arm Fractures

The systematic literature review resulted in five studies reporting incidence data on upper arm fractures with different locations. Three studies reported data on proximal humerus fractures, two studies on humerus shaft fractures, one study on the distal humerus and three studies on humerus fractures without specifying a more precise location. One study (Naranje et al., 2016 [11]) reported data on upper arm fractures, without further specifying a more precise location. The collected data are presented in Table 1.

In Romania and in the Romanian Western Region, the calculated incidences of upper arm fractures in children were 54.83/100,000 person-years and 64.79/100,000 person-years, respectively (Table 2).

### 3.2. Forearm Fractures

The systematic literature review resulted in nine studies reporting incidence data on forearm fractures with different locations. Three studies reported data on distal radius fractures, one study on radius/ulna fractures, one study on ulna shaft fractures, one study on radius shaft fractures and six studies on forearm fractures. Incidence values reported for the different locations are shown in Table 3.

In Romania and in the Romanian Western Region, the calculated incidences of forearm fractures in children were 139.77/100,000 person-years and 139.56/100,000 person-years, respectively (Table 4).

### 3.3. Upper Extremity Fractures

The systematic literature review resulted in one study reporting incidence data on upper extremity fractures without further specifying the location (Table 5).

In Romania and in the Romanian Western Region, the calculated incidences of upper extremity fractures (not otherwise specified) in children were 11.42/100,000 person-years and 15.79/100,000, respectively (Table 6).

The overall incidence of fractures calculated including all upper extremity locations is presented in Table 7.

## 4. Discussion

In Romania and in the Romanian Western Region, incidences of upper arm fractures in children were 54.83/100,000 person-years and 64.79/100,000 person-years, respectively, incidences of forearm fractures were 139.77/100,000 person-years and 139.56/100,000 person-years, respectively. Incidences of upper extremity fractures (not otherwise specified) were 11.42/100,000 person-years and 15.79/100,000, respectively.

The primary objective of our study was to collect recently published international incidence data on upper extremity fractures in children and to calculate incidence values based on data extracted from the centralized Romanian inpatient database. We found the data published in the scientific literature to be highly heterogeneous in terms of fracture locations.

Regarding the upper arm fractures, the study of Holloway et al. (2015) reported an incidence of 317.5 per 100,000 person-years in Australian children aged 0–9 years [12]. In Romania, with the patients included younger than 18 years, both nationwide and in the Western region, incidence was lower. The study of Christoffersen et al. (2016), performed in Norway, recorded an incidence of total radius and ulna fractures of 491 per 100,000 person-years [19]. In the same category of patients (younger than 18 years of age), the Romanian nationwide and Western region incidence was also lower. A possible explanation of the incidence values in Romania may be the fact that the incidence values were calculated using only inpatient data; emergency and ambulatory data were not included. This represents a limitation of the current study. A further limitation is that due to the heterogeneity of the data found in literature, we could not use the generally accepted 0–16 years of age interval and the open growth plates criterion for defining pediatric fractures.

Pasco et al., in an Australian study from 2015, reported an incidence peak in childhood and adolescence for both humerus and distal forearm fractures [14]. Higher humerus fracture peak incidence values were reported for both males and females than those in Romania for the same fracture location and similar age interval. When referring to the upper limb fractures, without a specific mention of the fracture site, the incidence in the USA ranged between 10 and 50 per 100,000 children-years [11]. In Romania, as well as in the Western part of the country, the incidence was similar. However, the incidence was reported per 100,000 person-years.

Since none of the relevant studies used diagnosis-based classification as location, a direct comparison of incidence values was not possible. Studies used different age intervals for reporting the incidence. The majority of the investigations used the standardized measurement reported to 100,000 person-years. Three studies mentioned annual incidence values per 1000 children (Wolfe et al., 2019 [13]; Naranje et al., 2016 [11]; Lyman et al., 2016 [20]), while other reported incidence per 100,000 hospital admissions per two years (Yang et al., 2019) [21].

The overall decreasing trend of the European pediatric population is continuing according to Eurostat projections for 2050–2100. In the long term, this will probably result in a decrease in the absolute number of fractures in children. However, the quantification of the impact of upper extremity fractures is necessary as this pathology has consequences on the children’s performance of activities of daily living, recreational and sport activities.

The incidence of upper extremity fractures should be regularly analyzed at both the national and regional levels in order to assure adequate healthcare personnel involved in the management of the affected children.

## 5. Conclusions

The incidence of upper extremity fractures in the pediatric population varies according to the analyzed data. Recently published epidemiological data in this field are scarce thus further studies are needed taking into account causes of trauma or mechanism of injury. Besides stratification by age, stratification by sex would also be important, since there are growth plate closure differences between girls and boys of the same age. The calculated incidence depends upon a variety of factors, such as the site of fractures, assessed population (worldwide, Romanian population or regional-Western part of Romania) or patients’ age.

## Figures and Tables

**Table 1:** Systematic literature review regarding the incidence values for upper arm fractures.

Fracture Location	First Author/Title	Study Country	Age (Years)/Age Interval	Gender	Incidence *
Humerus	Holloway K.L., Humeral Fractures in South-Eastern Australia: Epidemiology and Risk Factors [12]	Australia	0–9	Overall	**317.5**
	Wolfe J. A., Early Pediatric Fractures in a Universally Insured Population within the United States [13]	USA	<1	N/A	38 **
			1	N/A	112 **
			2	N/A	140 **
			3	N/A	164 **
			4	N/A	488 **
	Pasco J.A., The Epidemiology of Incident Fracture from Cradle to Senescence [14]	Australia	<20	Male	**232** **^†^**
				Female	**183** **^†^**
Humerus shaft	Holloway K.L., Humeral Fractures in South-Eastern Australia: Epidemiology and Risk Factors [12]	Australia	0–9	Overall	**294**
				Female	**298.8**
	Körner D., Change in paediatric upper extremity fracture incidences in German hospitals from 2002 to 2017: an epidemiological study [15]	Germany	0–4	Overall	2002: **4** 2017: **4**
			5–9	Overall	2002: **8** 2017: **5**
			10–14	Overall	2002: **10** 2017: **8**
			15–19	Overall	2002: **9** 2017: **6**
Proximal humerus	Holloway K.L., Humeral Fractures in South-Eastern Australia: Epidemiology and Risk Factors [12]	Australia	10–19	Male	**58.5**
	Hannonen J., The incidence and treatment trends of pediatric proximal humerus fractures [16]	Finland	<16	N/A	**31.4**
	Körner D., Change in paediatric upper extremity fracture incidences in German hospitals from 2002 to 2017: an epidemiological study [15]	Germany	0–4	Overall	2002: **3** 2017: **1**
			5–9	Overall	2002: **10** 2017: **6**
			10–14	Overall	2002: **23** 2017: **14**
			15–19	Overall	2002: **11** 2017: **7**
Distal humerus	Körner D., Change in paediatric upper extremity fracture incidences in German hospitals from 2002 to 2017: an epidemiological study [15]	Germany	0–4	Overall	2002: **48** 2017: **39**
			5–9	Overall	2002: **110** 2017: **102**
			10–14	Overall	2002: **43** 2017: **36**
			15–19	Overall	2002: **14** 2017: **9**
Upper arm	Naranje S.M., Epidemiology of Pediatric Fractures Presenting to Emergency Departments in the United States [11]	USA	0–19	Overall	30 ***
			<5		30 ***
			5–9		50 ***
			10–14		31 ***
			15–19		10 ***

* Incidence values are per 100,000 person-years, if not otherwise mentioned. ** Incidence values are per 100,000 children-years, values were transformed from annual incidence per 1000 children. *** Incidence values are per 100,000 children-years, values were transformed from annual incidence per 1000 children. ^†^ Incidence values are per 100,000 person-years; values were transformed from annual incidence per 10,000 person-years. N/A: not available.

**Table 2:** Incidence of upper arm fractures in Romania and in the Romanian Western Region.

Humerus Fractures	Total (Nationwide)	Western Region
Number of cases	2012	201
Population	3,669,563	310,254
Incidence	54.83	64.79

Incidence values are per 100,000 person-years.

**Table 3:** Systematic literature review regarding the incidence values for forearm fractures.

Fracture Location	First Author/Title	Study Country	Age (Years)/Age Interval	Gender	Incidence *
Ulna shaft	Körner D., Change in paediatric upper extremity fracture incidences in German hospitals from 2002 to 2017: an epidemiological study [15]	Germany	0–4	Overall	2002: **4** 2017: **5**
			5–9	Overall	2002: **10** 2017: **13**
			10–14	Overall	2002: **5** 2017: **4**
			15–19	Overall	2002: **3** 2017: **2**
Radius shaft	Körner D., Change in paediatric upper extremity fracture incidences in German hospitals from 2002 to 2017: an epidemiological study [15]	Germany	0–4	Overall	2002: **4** 2017: **4**
			5–9	Overall	2002: **13** 2017: **13**
			10–14	Overall	2002: **19** 2017: **15**
			15–19	Overall	2002: **9** 2017: **7**
Distal radius	Mamoowala N., Trends in paediatric distal radius fractures: an eight-year review from a large UK trauma unit [17]	UK	0–16	Overall	337 ***
			0–2	Overall	82 ***
			2–5	Overall	160 ***
			5–10	Overall	384 ***
				Male	381 ***
				Female	387 ***
			10–16	Overall	509 ***
	Hayashi S., Variation in fracture risk by season and weather: A comprehensive analysis across age and fracture site using a National Database of Health Insurance Claims in Japan [18]	Japan	0–19	Overall	**82.8**
			10–19	Overall	**212.4**
			0–9	Overall	**47.4**
	Körner D., Change in paediatric upper extremity fracture incidences in German hospitals from 2002 to 2017: an epidemiological study [15]	Germany	0–4	Overall	2002: **5** 2017: **4**
			5–9	Overall	2002: **45** 2017: **47**
			10–14	Overall	2002: **98** 2017: **92**
			15–19	Overall	2002: **45** 2017: **47**
Radius/ulna diaphyseal	Christoffersen T., Fracture incidence rates in Norwegian children, The Tromsø Study, Fit Futures [19]	Norway	<18	Overall	**19**
				Female	**26**
				Male	**13**
Radius/ulna distal				Overall	**439**
				Female	**423**
				Male	**456**
Radius/ulna proximal				Overall	**32**
				Female	**40**
				Male	**25**
Radius/ulna total				Overall	**491**
				Female	**489**
				Male	**494**
Forearm	Wolfe J. A., Early Pediatric Fractures in a Universally Insured Population within the United States [13]	USA	<1	N/A	56 **
			1	N/A	244 **
			2	N/A	245 **
			3	N/A	287 **
			4	N/A	856 **
	Naranje S.M., Epidemiology of Pediatric Fractures Presenting to Emergency Departments in the United States [11]	USA	0–19	Overall	**169**
			<5	Overall	**100**
			5–9	Overall	**252**
			10–14	Overall	**251**
			15–19	Overall	**78**
	Pasco J.A., The Epidemiology of Incident Fracture from Cradle to Senescence [14]	Australia	<20	Male	**170**
				Female	**125**
Forearm shaft	Körner D., Change in paediatric upper extremity fracture incidences in German hospitals from 2002 to 2017: an epidemiological study [15]	Germany	0–4	Overall	2002: **17** 2017: **31**
			5–9	Overall	2002: **57** 2017: **103**
			10–14	Overall	2002: **50** 2017: **72**
			15–19	Overall	2002: **13** 2017: **12**
Distal forearm	Lempesis V., Pediatric Distal Forearm Fracture Epidemiology in Malmö [5]	Sweden	N/A	Overall	**564**
				Male	**719**
				female	**401**
	Pasco J.A., The Epidemiology of Incident Fracture from Cradle to Senescence [14]	Australia	<20	Male	**948**
				female	**645**
	Körner D., Change in paediatric upper extremity fracture incidences in German hospitals from 2002 to 2017: an epidemiological study [15]	Germany	0–4	Overall	2002: **12** 2017: **9**
			5–9	Overall	2002: **65** 2017: **59**
			10–14	Overall	2002: **60** 2017: **53**
			15–19	Overall	2002: **11** 2017: **9**
Diaphyseal forearm	Lyman A., Pediatric diaphyseal forearm fractures: epidemiology and treatment in an urban population during a 10-year period, with special attention to titanium elastic nailing and its complications [20]	Sweden	0–16	Overall	70 **

* Incidence values are per 100,000 person-years, if not otherwise mentioned. ** Incidence values are per 100,000 children-years, values were transformed from annual incidence per 1000 children. *** Incidence values are per 100,000 children-years, as originally reported. NA: not available.

**Table 4:** Incidence of forearm fractures in Romania and in the Romanian Western Region.

Forearm Fractures	Total (Nationwide)	Western Region
Number of cases	5129	433
Population	3,669,563	310,254
Incidence	139.77	139.56

Incidence values are per 100,000 person-years.

**Table 5:** Systematic literature review regarding the incidence values for upper extremity fractures.

Fracture Location	First Author/Title	Study Country	Age (Years)/Age Interval	Gender	Incidence
Upper extremity	Yang H., Incidence patterns of traumatic upper limb fractures in children and adolescents Data from medical university-affiliated hospitals in Chongqing, China [21]	China	N/A	N/A	**101.6** (± **47.5**) *

* Incidence of traumatic upper limb fractures per 100,000 hospital admissions/2 years; N/A: not available.

**Table 6:** Incidence of upper extremity fractures in Romania and in the Romanian Western Region.

Upper Limb Fractures (Not Otherwise Specified)	Total (Nationwide)	Western Region
Number of cases	419	49
Population	3,669,563	310,254
Incidence	11.42	15.79

Incidence values are per 100,000 person-years.

**Table 7:** Overall incidence of upper extremity fractures (including upper arm, forearm and upper limb not otherwise specified) in Romania and in the Romanian Western Region.

Upper Extremity Fractures	Total (Nationwide)	Western Region
Number of cases	7560	683
Population	3,669,563	310,254
Incidence	206.02	220.14

Incidence values are per 100,000 person-years.
